# Estimation of Colistin Resistance Among Multidrug-Resistant Gram-Negative Bacilli: An Observational Study

**DOI:** 10.7759/cureus.108499

**Published:** 2026-05-08

**Authors:** Dolly Shiromani, Neelima Ranjan, Deepak Argal

**Affiliations:** 1 Microbiology, Gajra Raja Medical College, Gwalior, IND; 2 Dermatology, Government Medical College, Datia, IND

**Keywords:** antimicrobial resistance, broth micro dilution, colistin agar test, colistin broth disc elution (cbde), colistin resistance, gram negative bacteria (gnb), multidrug resistance (mdr)

## Abstract

Background: The rise in antimicrobial resistance, particularly among Gram-negative bacilli (GNB), poses a significant threat to global public health. Colistin, a polymyxin antibiotic, has re-emerged as a last-resort treatment against multidrug-resistant Gram-negative bacilli (MDR-GNB). However, increasing colistin resistance has become a major concern, especially in clinical settings where treatment options are limited.

Aim: To estimate the prevalence of colistin resistance among MDR-GNB and to compare the performance of three phenotypic methods, broth microdilution (BMD), colistin broth disk elution (CBDE), and colistin agar test (CAT), for detecting colistin resistance.

Methods: This observational study was conducted over a period of one year at a tertiary care hospital in Gwalior, Madhya Pradesh. A total of 100 MDR-GNB isolates were included as a convenience sample during the study period, which was considered adequate to estimate colistin resistance and evaluate the diagnostic performance of the testing methods.

The isolates were screened for colistin susceptibility using BMD (gold standard), CBDE, and CAT as per Clinical and Laboratory Standards Institute (CLSI) guidelines. Diagnostic performance, including sensitivity, specificity, and categorical agreement, was assessed for CBDE and CAT in comparison with BMD.

Results: Among 100 MDR Gram-negative isolates, *Klebsiella pneumoniae* (41%), *Escherichia coli* (35%), *Acinetobacter baumannii* (13%), and *Pseudomonas aeruginosa* (11%) were identified. Susceptibility to imipenem was highest in *E. coli* (20/35; 57.1%), followed by *P. aeruginosa* (6/11; 54.5%) and *A. baumannii* (7/13; 53.8%), while *K. pneumoniae* showed lower susceptibility (13/41; 31.7%). Resistance to ciprofloxacin was observed in 100% of *K. pneumoniae*, *P. aeruginosa*, and *A. baumannii*, and 94.3% (33/35) of *E. coli*. Resistance to third-generation cephalosporins ranged from 81.8% to 100%.

Colistin resistance by BMD was 4% (4/100), with resistance detected in *K. pneumoniae* (3/41; 7.3%) and *A. baumannii* (1/13; 7.7%). The CBDE method showed 100% sensitivity, 98.96% specificity, and 99% categorical agreement with BMD, whereas the CAT demonstrated lower sensitivity (75%) and a higher very major error rate (25%).

Conclusion: The emergence of colistin-resistant MDR-GNB emphasizes the urgent need for accurate and accessible susceptibility testing. While BMD remains the reference standard, CBDE offers a practical and reliable alternative for routine laboratory use. Regular surveillance and rational use of colistin are essential to curb the spread of resistance and preserve the efficacy of this critical antimicrobial agent.

## Introduction

The emergence of antibiotic resistance among Gram-negative bacteria since the 1970s has become a critical global crisis [1]. India had the highest antibiotic consumption, followed by China and the United States, with an overall increase of 36% [2]. Between 2000 and 2015, antibiotic consumption in India increased by 103% [3].

In India, members of Enterobacteriaceae, *Pseudomonas aeruginosa*, and *Acinetobacter baumannii* are common clinical pathogens, with rising extended-spectrum beta-lactamase (ESBL) production and carbapenem resistance posing a serious concern. The prevalence of resistance to polymyxins has been estimated at 15.0% in India, which is higher than the global average [4]. Approved by the United States Food and Drug Administration (US FDA) in 1959, colistin is used as a last-resort bactericidal agent against multidrug-resistant (MDR) Gram-negative pathogens such as *P. aeruginosa, A. baumannii, Escherichia coli, *and* Klebsiella pneumoniae* [5]. The main concern with colistin use is determining the correct dosage and frequency to ensure bactericidal activity against MDR bacteria, especially given the varying breakpoints assigned to different species.

Till 2019, broth microdilution (BMD) was the only option available for antimicrobial susceptibility testing (AST) of colistin, but since 2020, the Clinical and Laboratory Standards Institute (CLSI) has also approved colistin broth disk elution (CBDE) and colistin agar test (CAT) [6]. BMD could not be implemented routinely, as it is a labour-intensive procedure, whereas both CBDE and CAT can be used for testing colistin susceptibility in clinical diagnostics, as they are relatively easy to perform [7,8]. In light of the growing threat posed by multidrug-resistant Gram-negative bacilli (MDR-GNB), the present study was undertaken to evaluate the prevalence of colistin resistance in our clinical setting. Additionally, the study aimed to compare the diagnostic performance of various phenotypic methods for colistin susceptibility testing, with a focus on identifying reliable and practical alternatives to the gold standard BMD technique.

## Materials and methods

This observational study was conducted in the Department of Microbiology at a tertiary healthcare center from January 2024 to January 2025. A sample size of 100 MDR-GNB isolates was taken based on the availability of isolates during the study period. It was considered adequate to assess colistin resistance and to compare the performance of the different testing methods used in the study. A total of 100 MDR-GNB isolates were collected from various clinical samples (urine, blood, pus, cerebrospinal fluid (CSF), throat swabs, sputum, and other body fluids) from both the Outpatient Department (OPD) and the Inpatient Department (IPD) patients. The study was approved by the Institutional Human Ethics Committee (Approval No. 75/IEC-GRMC/2023). Inclusion criteria focused on properly collected samples and MDR Enterobacterales or non-fermenters resistant to ≥3 antimicrobial classes, excluding those intrinsically resistant to colistin. Exclusion criteria included delayed or improperly collected samples and isolates sensitive to >3 antimicrobial classes.

Statistical analysis

Data were analyzed using Microsoft Excel (Microsoft, Redmond, WA, USA) and open-source software jamovi (version 2.6; The jamovi Project, 2025) for statistical analysis. BMD was used as the reference method for the detection of colistin susceptibility.

Laboratory methods

For the identification and isolation of 100 MDR-GNB, conventional methods for bacterial identification were used. First, direct Gram staining was done from the sample, and thereafter, samples were inoculated on blood agar and MacConkey agar as per standard protocol and incubated at 37°C for 24 h.

Gram staining, motility testing (hanging drop), and a series of standard biochemical tests were used for isolating GNB. Rapid tests included the oxidase test, catalase test, and motility test. Other tests performed were the oxidation-fermentation test, indole production test, urease hydrolysis test, Simmons citrate utilization test, triple sugar iron agar test, and mannitol motility test. No automated culture system was used in this study. Antimicrobial susceptibility testing was done by the Kirby-Bauer disk diffusion method. For different bacterial isolates, different antimicrobial panels were used for AST following CLSI M100 guidelines [8].

**Figure 1:**
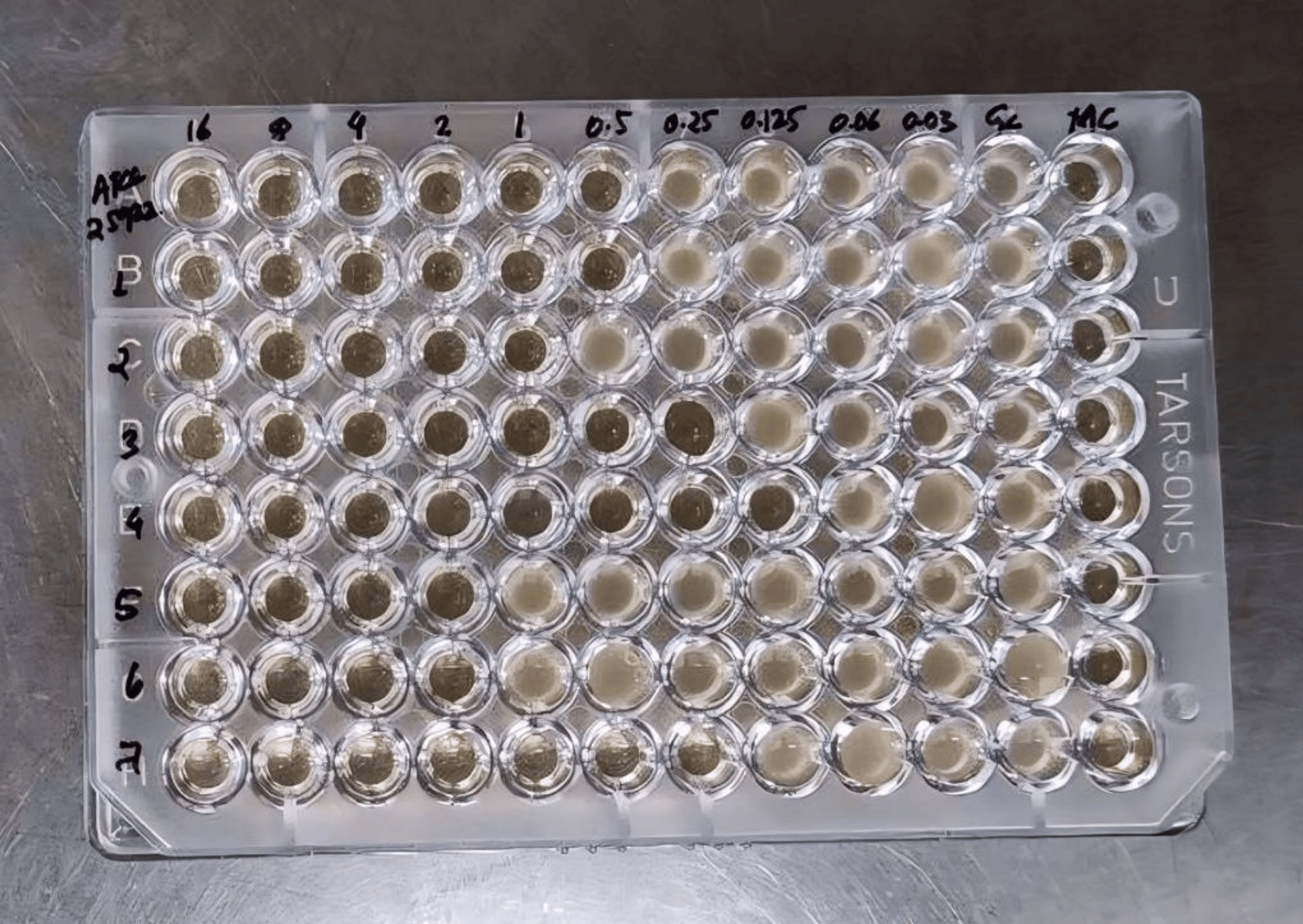
Colistin BMD BMD: broth microdilution

Colistin resistance was assessed using three different methods. The first method was BMD, the gold standard method, which was performed using cation-adjusted Mueller-Hinton broth (CAMHB). The source of Mueller-Hinton Broth (MHB No. 2, cation-adjusted) was HIMEDIA Laboratories. Then, a stock solution of colistin was prepared by calculating the potency of colistin sulfate powder (more than 19000 U/mg). After that, a working stock solution of 4× final drug concentration was prepared. Dilutions of colistin were made to achieve the final concentration in microtiter plate wells. Inoculum was prepared by adjusting the turbidity to 0.5 McFarland and then diluting it 1:75 times. Twenty-five μL of this suspension was added with 50 μL MHB and 25 μL drug solution to each well of the microtiter plate. The microtiter plates were incubated at 35°C for 16 to 24 h (Figure 1). For quality control, the ATCC strain used for testing against Enterobacteriaceae was ATCC 25922, and for *P. aeruginosa*, ATCC 27853 was used.

**Figure 2:**
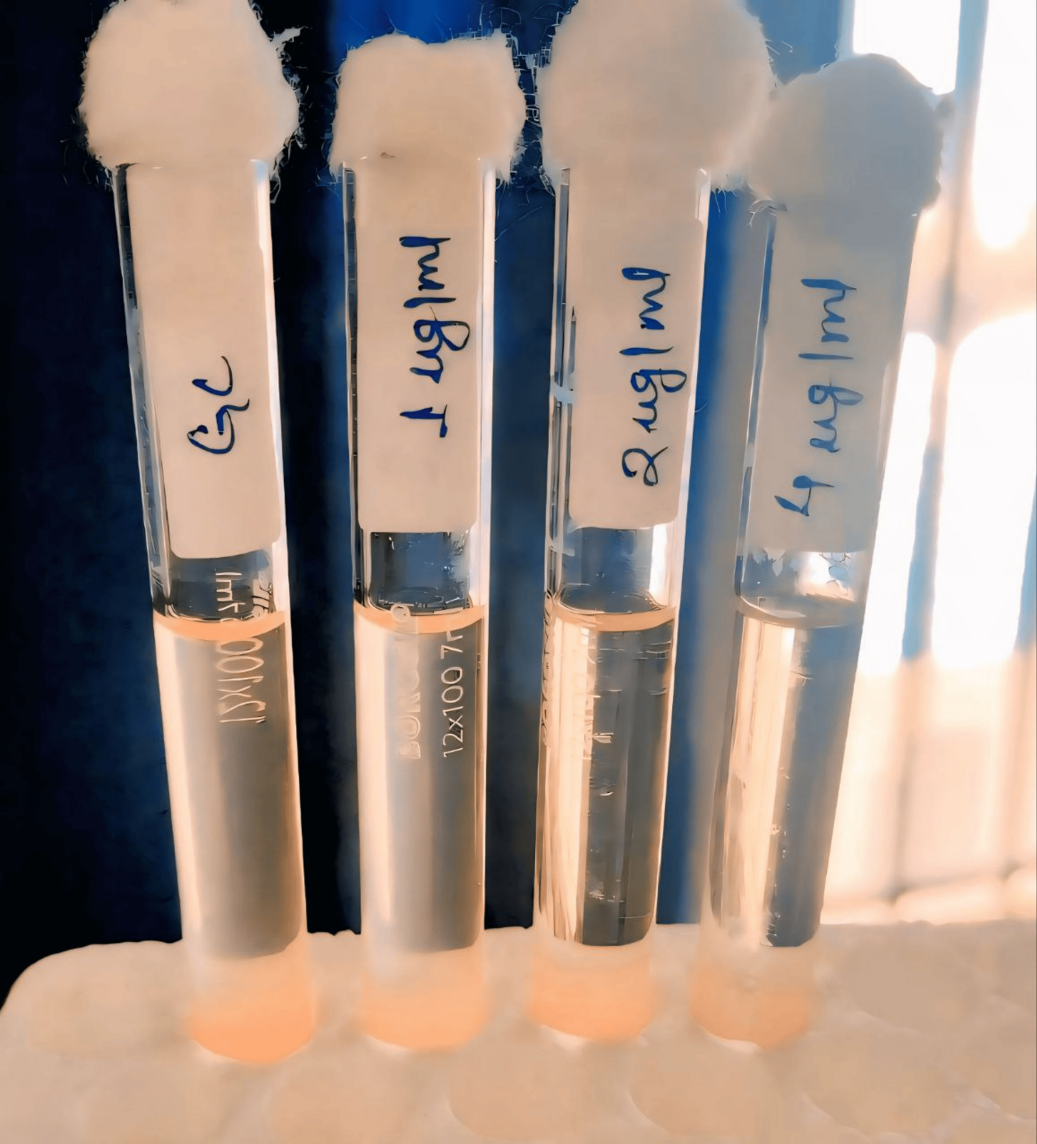
CBDE determination CBDE: colistin broth disk elution

The second method was CBDE. For each isolate, four test tubes were used and labeled as growth control (GC), 1, 2, and 4. Each tube was filled with 10 mL of CAMHB sourced from HIMedia Laboratories (HiMedia Laboratories Pvt. Ltd., Mumbai, India). Colistin discs (TM media, India), each containing 10 µg of colistin, were used. A total of seven discs were required per isolate. The first tube was labelled as GC, in which no colistin disc was added. In tube 1 (1 µg/mL), one colistin disc was added; in tube 2 (2 µg/mL), two colistin discs were added; and in tube 4 (4 µg/mL), four colistin discs were added. Then, 50 µL of the inoculum was added to each of the four tubes and incubated at 37°C for 20-24 hrs. The minimum inhibitory concentration (MIC) was read as the lowest concentration without visible growth (Figure 2).

**Figure 3:**
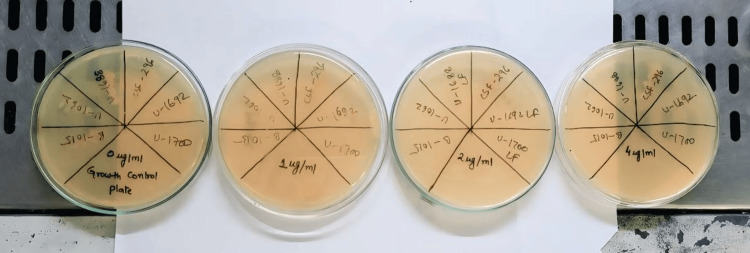
Mueller-Hinton agar plates with colistin concentrations of 0 μg/mL, 1 μg/mL, 2 μg/mL, and 4 μg/mL used for CAT CAT: colistin agar test

The third method used was CAT, which was performed using colistin sulfate powder with a potency of more than 19000 U/mg sourced from HIMEDIA. Mueller-Hinton agar plates with colistin concentrations of 0 μg/mL, 1 μg/mL, 2 μg/mL, and 4 μg/mL were prepared. After that, 10 μL of the 1:10 dilution of each isolate was streaked, and the plates were incubated at 35°C for 18-20 hrs (Figure 3). MIC was defined as the lowest concentration of colistin that inhibited visible bacterial growth.

## Results

The present study, conducted in the Department of Microbiology, analyzed patient data to meet its objectives. The age distribution of the study population (n=100) showed that the majority of patients were in the younger age groups, with 31% each in the ≤20 years and 21-40 years categories. This was followed by 26% in the 41-60 years group, while the least proportion (12%) was observed in patients aged 61-80 years (Table 1).

**Table 1:** Age-wise distribution of patients with MDR-GNB isolates (n=100) MDR-GNB: multidrug-resistant Gram-negative bacilli

Age group	Number (percentage %)
≤20 Years	31 (31)
21-40 Years	31 (31)
41-60 Years	26 (26)
61-80 Years	12 (12)
Total	100 (100)

The most common provisional diagnosis was urinary tract infection (UTI), observed in 28 (28%) cases, followed by sepsis in 27 (27%), abscess in 22 (22%), meningitis in 10 (10%), pneumonia in eight (8%), and pyrexia of unknown origin in five (5%) patients (Table 2).

**Table 2:** Distribution of patients according to provisional diagnosis (n=100) PUO: pyrexia of unknown origin; UTI: urinary tract infection

Provisional diagnosis	Number/percentage (%)
Abscess	22 (22)
Meningitis	10 (10)
Pneumonia	8 (8)
PUO	5 (5)
Sepsis	27 (27)
UTI	28 (28)
Total	100 (100)

The antimicrobial susceptibility pattern of MDR Gram-negative isolates showed variable activity across antibiotics. Susceptibility to imipenem was highest in *E. coli* (20/35; 57.1%), followed by *P. aeruginosa* (6/11; 54.5%) and* A. baumannii* (7/13; 53.8%), while *K. pneumoniae *demonstrated lower susceptibility (13/41; 31.7%). Doxycycline exhibited moderate susceptibility, ranging from 30.8% to 45.5% across isolates. Resistance to ciprofloxacin was nearly universal, observed in 100% of *K. pneumoniae, P. aeruginosa*, and *A. baumannii*, and in 94.3% (33/35) of *E. coli*. Similarly, resistance to third-generation cephalosporins (ceftriaxone and ceftazidime) ranged from 81.8% to 100%. Piperacillin-tazobactam showed limited susceptibility (7.7%-18.2%). Among urinary* E. col*i isolates tested for nitrofurantoin (n=23), susceptibility was observed in 21.7% (5/23) of cases (Table 3).

**Table 3:** Organism-wise antimicrobial susceptibility pattern of MDR gram-negative isolates (n=100) ^1^ Ampicillin: Intrinsic resistance in *K. pneumoniae* and non-fermenters. ^2^ Ceftriaxone: Not applicable for *P. aeruginosa*. ^3^ Ceftazidime: Resistance may be due to ESBL or other β-lactamase production. ^4^ Amoxicillin-clavulanate: Not effective against *P. aeruginosa*; limited activity in *A. baumannii*. ^5^ Nitrofurantoin: Tested only in urinary isolates (*E. coli*, n=23). ^* ^Percentages calculated based on tested isolates (n=23), not total (n=35). R indicates that the isolate was resistant to the tested antibiotic. S indicates that the isolate was susceptible to the tested antibiotic. MDR: multidrug-resistant; ESBL: extended-spectrum beta-lactamase, R: resistant; S: susceptible

Antibiotic	*Klebsiella pneumoniae* (n=41), R/S, n (%)	*Escherichia coli* (n=35), R/S, n (%)	*Pseudomonas aeruginosa *(n=11), R/S, n (%)	*Acinetobacter baumannii* (n=13), R/S, n (%)
Amikacin	29 (70.7%)/12 (29.3%)	24 (68.6%)/11 (31.4%)	8 (72.7%)/3 (27.3%)	9 (69.2%)/4 (30.8%)
Ampicillin^1^	NA	35 (100%)/0 (0%)	NA	NA
Ceftriaxone^2^	41 (100%)/0 (0%)	29 (82.9%)/6 (17.1%)	NA	13 (100%)/0 (0%)
Doxycycline	25 (61.0%)/16 (39.0%)	24 (68.6%)/11 (31.4%)	6 (54.5%)/5 (45.5%)	9 (69.2%)/4 (30.8%)
Imipenem	28 (68.3%)/13 (31.7%)	15 (42.9%)/20 (57.1%)	5 (45.5%)/6 (54.5%)	6 (46.2%)/7 (53.8%)
Gentamicin	36 (87.8%)/5 (12.2%)	29 (82.9%)/6 (17.1%)	8 (72.7%)/3 (27.3%)	10 (76.9%)/3 (23.1%)
Ciprofloxacin	41 (100%)/0 (0%)	33 (94.3%)/2 (5.7%)	11 (100%)/0 (0%)	13 (100%)/0 (0%)
Ceftazidime^3^	41 (100%)/0 (0%)	35 (100%)/0 (0%)	9 (81.8%)/2 (18.2%)	12 (92.3%)/1 (7.7%)
Amoxicillin-clavulanate^4^	41 (100%)/0 (0%)	35 (100%)/0 (0%)	NA	13 (100%)/0 (0%)
Nitrofurantoin^5^	NA	18 (78.3%)/5 (21.7%)^*^	NA	NA
Piperacillin-tazobactam	37 (90.2%)/4 (9.8%)	29 (82.9%)/6 (17.1%)	9 (81.8%)/2 (18.2%)	12 (92.3%)/1 (7.7%)

**Figure 4:**
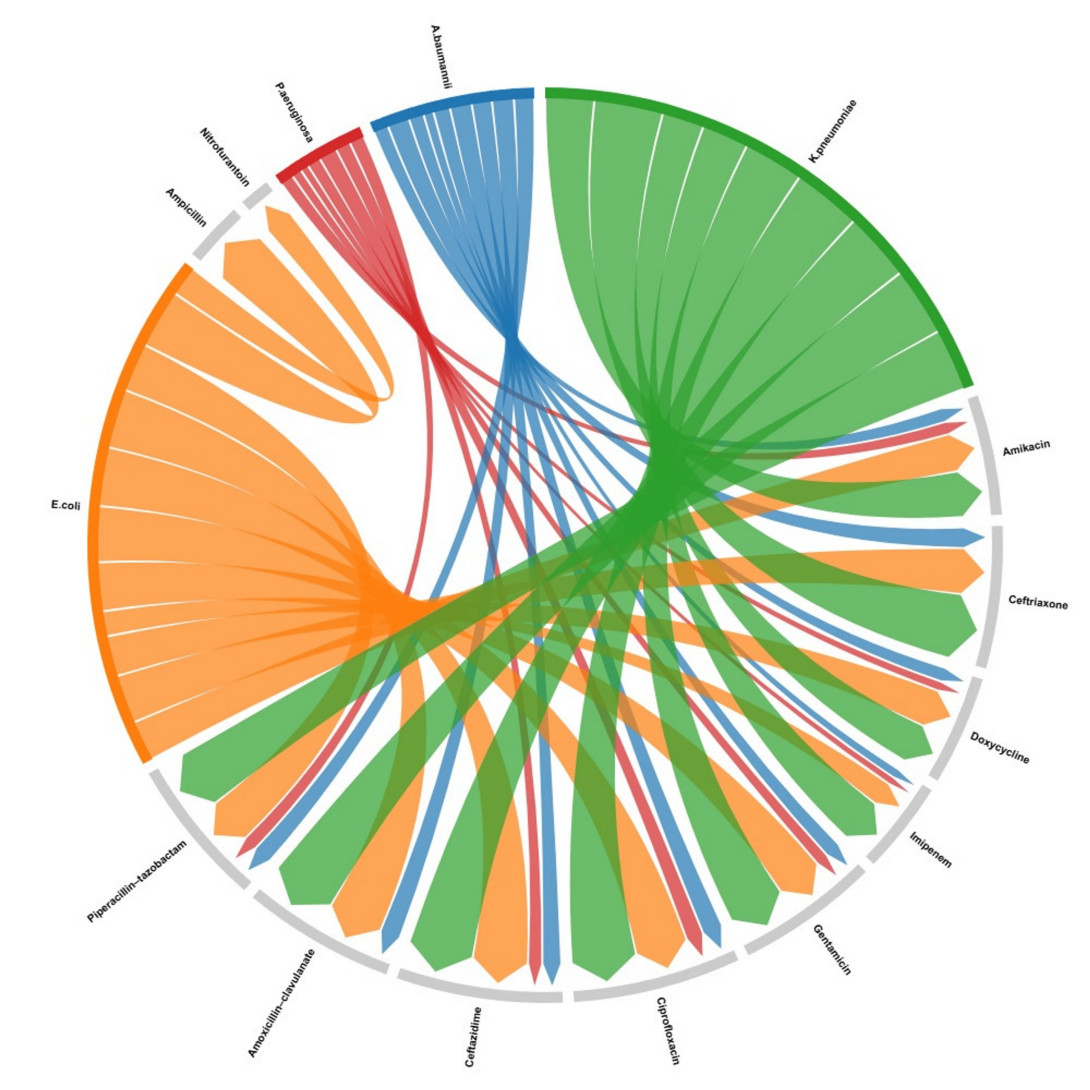
Relationship between Gram-negative bacteria and antibiotic resistance: a chord diagram Chord diagram showing antibiotic resistance patterns among MDR-GNB. The width of each chord indicates the number of resistant isolates, with only relevant organism-antibiotic combinations included. MDR-GNB: multidrug-resistant Gram-negative bacilli Image was created using RAWGraphs (DensityDesign Research Lab, Politecnico di Milano, Milan, Italy).

The resistance pattern of MDR Gram-negative isolates demonstrated variability across the tested antibiotics (Figure 4). Imipenem showed the lowest resistance at 54% (54/100), followed by doxycycline (64%; 64/100) and amikacin (70%; 70/100). Resistance to ciprofloxacin was observed in 98% (98/100) of isolates. Among β-lactams, ceftriaxone resistance was 93% (83/89 tested isolates) and ceftazidime resistance was 97% (97/100). Amoxicillin-clavulanate and ampicillin demonstrated 100% resistance among tested isolates (76/76 and 35/35, respectively). Piperacillin-tazobactam resistance was 87% (87/100), while gentamicin resistance was 83% (83/100). Among urinary isolates, nitrofurantoin resistance was 78.3% (18/23). Overall, resistance across antibiotics ranged from 54% to 100%, with relatively lower resistance observed for imipenem compared to other agents (Table 4).

**Table 4:** Organism-wise antibiotic resistance pattern of MDR Gram-negative isolates (n=100) ^1^Ampicillin: Intrinsic resistance in *K. pneumoniae* and non-fermenters. ^2^Ceftriaxone: Not applicable for* P. aeruginosa.* ^3^Ceftazidime: Resistance may be due to ESBL or other β-lactamase production. ^4^Amoxicillin-clavulanate: Not effective against *P. aeruginosa*; limited activity in *A. baumannii*. ^5^Nitrofurantoin: Tested only in urinary isolates (*E. coli*, n=23). ^*^Percentages calculated based on tested isolates (n=23), not total (n=35). MDR: multidrug-resistant; ESBL: extended-spectrum beta-lactamase

Antibiotic	*Klebsiella pneumoniae *(n=41), n (%)	*Escherichia coli* (n=35), n (%)	*Pseudomonas aeruginosa *(n=11), n (%)	*Acinetobacter baumannii *(n=13), n (%)
Amikacin	29 (70.7%)	24 (68.6%)	8 (72.7%)	9 (69.2%)
Ampicillin¹	NA	35 (100%)	NA	NA
Ceftriaxone²	41 (100%)	29 (82.9%)	NA	13 (100%)
Doxycycline	25 (61.0%)	24 (68.6%)	6 (54.5%)	9 (69.2%)
Imipenem	28 (68.3%)	15 (42.9%)	5 (45.5%)	6 (46.2%)
Gentamicin	36 (87.8%)	29 (82.9%)	8 (72.7%)	10 (76.9%)
Ciprofloxacin	41 (100%)	33 (94.3%)	11 (100%)	13 (100%)
Ceftazidime³	41 (100%)	35 (100%)	9 (81.8%)	12 (92.3%)
Amoxicillin-clavulanate⁴	41 (100%)	35 (100%)	NA	13 (100%)
Nitrofurantoin⁵	NA	18 (78.3%)^*^	NA	NA
Piperacillin-tazobactam	37 (90.2%)	29 (82.9%)	9 (81.8%)	12 (92.3%)

The distribution of colistin MIC values in both CBDE and CAT was shown. In both methods, MIC of 1 µg/mL was the most frequent, followed by MIC of 2 µg/mL, while higher MIC values (4 µg/mL) were rare. A slightly higher proportion of isolates at MIC 2 µg/mL was observed with the CAT (Table 5).

**Table 5:** Distribution of colistin MICs among MDR-GNB by testing method MIC values are expressed in µg/mL. Percentages are calculated based on the total number of isolates for each organism. CAT: colistin agar test; CBDE: colistin broth disk elution; MIC: minimum inhibitory concentration; MDR-GNB: multidrug-resistant Gram-negative bacilli

MIC (µg/mL)	Method	*Klebsiella pneumoniae* (n=41)	*Escherichia coli* (n=35)	*Pseudomonas aeruginosa* (n=11)	*Acinetobacter baumannii *(n=13)
1	CBDE	29 (70.7%)	32 (91.4%)	11 (100%)	10 (76.9%)
	CAT	28 (68.3%)	30 (85.7%)	9 (81.8%)	10 (76.9%)
2	CBDE	9 (22.0%)	3 (8.6%)	0	1 (7.7%)
	CAT	11 (26.8%)	5 (14.3%)	2 (18.2%)	3 (23.1%)
4	CBDE	3 (7.3%)	0	0	2 (15.4%)
	CAT	2 (4.9%)	0	0	0

Based on BMD, 4% of isolates were resistant to colistin (>2 µg/mL), including *K. pneumoniae* (7.3%) and *A. baumannii *(7.7%), while all *E. coli* and *P. aeruginosa* isolates were susceptible (MIC ≤2 µg/mL). The CBDE method showed comparable results, detecting 5% resistance overall, with resistance observed in *K. pneumoniae* (7.3%) and *A. baumannii* (15.4%). In contrast, the CAT detected only 2% resistance, limited to *K. pneumoniae* (4.9%). Overall susceptibility (MIC ≤2 µg/mL) was high across all methods: 96% by BMD, 95% by CBDE, and 98% by CAT. CBDE demonstrated high agreement with BMD, whereas CAT showed lower detection of resistant isolates (Table 6).

**Table 6:** Organism-wise distribution of colistin MICs among MDR-GNB using CBDE, CAT, and BMD methods (n=100) Interpretation based on CLSI guidelines: MIC ≤2 µg/mL indicates intermediate, and MIC >2 µg/mL indicates resistant. MDR-GNB: multidrug-resistant Gram-negative bacilli; CAT: colistin agar test; CBDE: colistin broth disk elution; MIC: minimum inhibitory concentration; CLSI: Clinical and Laboratory Standards Institute

Method	MIC (µg/mL)	*Klebsiella pneumoniae *(n=41)	*Escherichia coli* (n=35)	*Pseudomonas aeruginosa *(n=11)	*Acinetobacter baumannii* (n=13)
CBDE	≤2	38 (92.7%)	35 (100%)	11 (100%)	11 (84.6%)
	>2	3 (7.3%)	0 (0%)	0 (0%)	2 (15.4%)
CAT	≤2	39 (95.1%)	35 (100%)	11 (100%)	13 (100%)
	>2	2 (4.9%)	0 (0%)	0 (0%)	0 (0%)
BMD	≤2	38 (92.7%)	35 (100%)	11 (100%)	12 (92.3%)
	>2	3 (7.3%)	0 (0%)	0 (0%)	1 (7.7%)

Using BMD as the reference method, the CBDE method demonstrated high diagnostic accuracy with sensitivity and specificity of 100.0% and 98.96%, respectively, and a categorical agreement of 99.0%. The CAT showed comparatively lower sensitivity (75.0%) with similar specificity (98.96%) and a categorical agreement of 98.0%. A higher very major error rate was observed with the CAT (25.0%) compared to no very major errors in the CBDE method (Table 7).

**Table 7:** Performance evaluation of colistin susceptibility testing methods using BMD as reference (n=100) Performance of CBDE and CAT was evaluated in comparison with BMD as the reference standard. BMD: broth microdilution; CBDE: colistin broth disk elution; CAT: colistin agar test; TP: true positive; TN: true negative; FP: false positive; FN: false negative; ME: major error; VME: very major error; CA: categorical agreement; PPV: positive predictive value; NPV: negative predictive value

Parameters	BMD (Reference)	CBDE	CAT
TP	4	4	3
FN	0	0	1
FP	0	1	1
TN	96	95	95
ME %	-	1.04	1.04
VME %	-	0.00	25.00
CA %	100.00	99.00	98.00
Sensitivity (%)	100.00	100.00	75.00
Specificity (%)	100.00	98.96	98.96
PPV %	100.00	80.00	75.00
NPV %	100.00	100.00	98.96

## Discussion

This observational study assessed colistin resistance in MDR-GNB and evaluated susceptibility testing methods. Among 100 MDR-GNB isolates obtained from patients of different age groups, no significant age-wise correlation was observed, consistent with previous findings, highlighting the need for universal infection control measures irrespective of age [9].

UTIs (28%) were the most common provisional diagnosis, followed by sepsis (27%), consistent with global trends where *E. coli*-associated UTIs are highly prevalent [10]. Blood (32%), urine (28%), and pus (22%) were the most common specimens received [11,12]. *K. pneumoniae* (41%) and *E. coli *(35%) were the predominant MDR-GNB isolates, aligning with global surveillance data identifying them as major drivers of MDR infections [13,14].

The resistance pattern in MDR-GNB showed that ciprofloxacin resistance was observed in 98% of isolates, consistent with global reports [14,15]. Imipenem resistance (54%) indicates increasing carbapenem resistance, similar to findings by Patrice Nordmann et al. [16]. Doxycycline showed moderate activity with 36% susceptibility, comparable to previous studies [17].

Despite widespread resistance, colistin remained highly active, with 96% susceptibility by BMD. CBDE showed strong agreement with BMD (99% categorical agreement, 100% sensitivity, and 98.96% specificity), supporting its reliability as a cost-effective method [18,19]. In contrast, CAT showed lower performance due to poor cationic molecule diffusion and low sensitivity in detecting resistance, which may miss some colistin-resistant isolates. In our study, CAT also showed lower performance, with 75% sensitivity and a 25% very major error (VME) rate, consistent with previous reports [20].

Most *K. pneumoniae* and *E. coli *isolates showed colistin MICs of 1 µg/mL, particularly with CBDE, indicating preserved susceptibility. Overall, 96% of MDR isolates had MIC ≤2 µg/mL by BMD, while 4% had MIC ≥4 µg/mL. These results align with previous studies reporting MIC clustering around 1-2 µg/mL with method-dependent variability [20,21].

BMD detected colistin resistance in 4% of isolates, with higher rates in *A. baumannii* and K. pneumoniae, consistent with Humphries et al. [18]. CBDE showed strong agreement with BMD (100% sensitivity, 98.96% specificity, and 99% agreement) [19]. In contrast, CAT detected only 2% resistance with lower sensitivity (75%) and a high VME rate, as reported by Jayol et al. [21]. Overall, BMD remains the gold standard, while CBDE is a reliable alternative, and CAT should be used cautiously.

The emergence of colistin resistance in Gram-negative pathogens such as *P. aeruginosa,* *A. baumannii*, and *K. pneumoniae* has become a global concern in recent years [22]. A previous study showed 57% and 22% colistin resistance rates in *K. pneumoniae* and *P. aeruginosa* isolates, respectively [23].

The emergence of colistin heteroresistance is a public health concern at both diagnostic and treatment levels. The presence of clinically undetected colistin-heteroresistant subpopulations leads to treatment failure, prolonged hospital stays, and even death of the patient [24]. Therefore, there is a need to raise awareness among microbiologists and specialists regarding colistin heteroresistance mechanisms to achieve effective treatment.

Limitations

Being a single-center study with a small sample size (100 isolates), the findings may not be generalizable to other regions. Molecular methods, such as polymerase chain reaction (PCR) for detecting resistance genes (e.g., mcr-1 and mcr-2), were not performed, limiting the genetic insights. Manual media preparation may have introduced variability, and only phenotypic methods were used, which might have missed heteroresistance or low-level resistance.

## Conclusions

Colistin resistance among MDR Gram-negative isolates, although low, remains a clinically important concern. For years, reliable susceptibility testing for colistin was limited to labor-intensive methods like BMD, discouraging routine use. Thus, the study effectively met its goals by quantifying the prevalence of colistin resistance in MDR-GNB and establishing CBDE as a dependable and feasible option for routine susceptibility testing, particularly for laboratories with limited infrastructure, where adopting accurate but less resource-intensive methods is crucial for guiding appropriate antimicrobial therapy.

The CBDE method demonstrated high reliability compared to the reference BMD method and can be used as a cost-effective alternative for routine testing, while the CAT should be interpreted with caution.
